# Delivery of Psychoeducation for Supporting Brain Health in Aging: Across People, Places, and Spaces

**DOI:** 10.1093/geroni/igaf122.1113

**Published:** 2025-12-31

**Authors:** Emily Trittschuh, Sarah Punshon, Mary Wyman

## Abstract

There is an increased demand for information on how to support brain health in aging. Research has identified a number of healthy lifestyle behaviors and treatable health conditions that are supportive – and might help prevent dementia. Translating research study findings into actionable behavioral change for the layperson is important. This symposium was developed to highlight four health education programs which attempt to address this need, and which do so across different populations, in different settings and for unique combinations of these. AgeWISE is a 12-week highly manualized brain health education program that is rich in content developed for older adults with intact cognitive abilities and high drive to engage. Healthy Aging Project-Brain (HAP-B) is an ambulatory outpatient psychoeducational group that is less strictly manualized and only 6 sessions, developed for older Veterans and successfully deployed both in-person and via clinical video technology. Healthy Aging Project-Brain, Occupational Therapy (HAP-B OT) is designed for implementation with folks in subacute rehab, residential and/or other inpatient settings with a focus on independent sessions that can be tailored to a wider range of cognitive and physical limitations. In the UW Madison and Madison VA rural dementia care telehealth initiative, seminars on healthy brain aging are provided in rural communities to support community building and successful implementation of the telehealth initiative. This symposium will describe the development of these interventions, their evolution to serve different populations while maintaining fidelity, and the ongoing translation and dissemination of the interventions into different healthcare and community environments.

